# Microbiology of bloodstream infections in Ontario, Canada during COVID-19 pandemic

**DOI:** 10.14745/ccdr.v50i34a05

**Published:** 2024-04-30

**Authors:** Mohammad R Hasan, Yasmeen M Vincent, Daniela Leto, Huda Almohri

**Affiliations:** 1 Medical and Scientific Department, LifeLabs, Toronto, ON; 2 Department of Pathology and Molecular Medicine, McMaster University, Hamilton, ON

**Keywords:** bloodstream infections, Ontario, COVID-19, blood culture, microbiology

## Abstract

**Background:**

Bloodstream infections (BSI) caused by a wide range of bacterial and fungal pathogens are associated with high rates of morbidity and mortality. Based on an estimate in 2017, the number of BSI incidences in Ontario is 150 per 100,000 population. The epidemiology of BSIs may be affected by many factors, including the social and travel restrictions and increased rates of hospitalizations in Ontario during the coronavirus disease 2019 (COVID-19) pandemic.

**Objectives:**

This study aimed to assess the changes in the microbiology of BSIs in Ontario during the COVID-19 pandemic compared to the pre-pandemic period.

**Methods:**

Retrospective blood culture data (n=189,106) from LifeLabs Ontario (July 2018 to December 2021) were analyzed. Blood culture positivity rates for common bacterial pathogens were compared between pre-COVID-19 (July 2018 to March 2020) and COVID-19 (April 2020 to December 2021) periods in community and hospital settings, using the chi-square test for significance.

**Results:**

During the COVID-19 period, blood culture positivity rates in the community remained the same, while hospital rates increased by approximately threefold (*p*=0.00E-00). In the community, the isolation rates of most bacterial species remained unchanged, except for an increase in *Enterococcus* spp. and a decrease in *Salmonella* spp. The rates of antibiotic-resistant organisms (AROs) also significantly decreased in the community. In hospitals, all bacterial species, including AROs, showed significant increases in isolation rates during the COVID-19 period.

**Conclusion:**

The study revealed shifts in the microbiology of BSIs and suggests changes in the epidemiology of BSIs during the COVID-19 pandemic in Ontario, both in hospitals and in the community.

## Introduction

Bloodstream infections (BSIs) have a considerable impact on healthcare settings and communities because of high rates of morbidity and mortality associated with such infections ((1)). In hospitals, they are among the most common healthcare-associated infections. Studies have reported varying incidence rates, ranging from 1.5 to 4.0 cases per 1,000 patient days. The incidence of community-acquired BSIs is lower but still significant, affecting individuals outside of healthcare facilities ((2)). In Ontario, based on a population-wide retrospective cohort study of BSIs in 2017, there were 150 BSI episodes per 100,000 population with a 30-day mortality rate of 17% ((3)).

The causative agents of BSIs vary depending on the setting, patient population, and regional factors. Gram-positive bacteria are commonly implicated, with *Staphylococcus aureus*, including methicillin-resistant strains (MRSA), being a leading cause. Coagulase-negative staphylococci, such as *Staphylococcus epidermidis*, are also frequently isolated. Gram-negative bacteria, including *Escherichia coli*, *Klebsiella pneumoniae*, and *Pseudomonas aeruginosa*, contribute significantly to BSIs, particularly in healthcare settings. Fungal pathogens, such as *Candida* spp., are an important cause of BSIs in immunocompromised individuals. The emergence and spread of antimicrobial resistance pose additional challenges in managing BSIs. Methicillin-resistant *Staphylococcus aureus* and extended-spectrum beta-lactamase (ESBL) producing gram-negative bacteria have been associated with increased mortality and healthcare costs ((1,3)).

The epidemiology of BSIs has been changing in recent decades, driven by many factors, such as changing population demographics, healthcare delivery methods, and increasing globalization ((1)). Most recently, BSI epidemiology in the community and hospitals may have been impacted by mobility restrictions and increased rates of hospitalizations associated with coronavirus disease 2019 (COVID-19). In this study, we assessed the microbiology of BSIs in Ontario during the COVID-19 pandemic and compared it to the pre-pandemic period.

## Methods

In this retrospective observational study, data from blood cultures (n=189,106) performed by LifeLabs medical laboratories in Ontario from July 2018 to December 2021 were utilized; the cultures were collected from patients attending primary care facilities and 36 hospitals across the province. For hospitals, more than 90% of blood cultures were from five general community hospitals in the Hamilton Niagara Haldimand Brant Local Health Integration Network (LHIN) that have 100 or more patient beds. For the blood cultures from communities, more than 70% were from urban communities. Data were retrieved without any patient identifying information, according to the LifeLabs code of ethics policy. Blood culture positivity rates for all pathogens and for most frequently isolated bacterial pathogens were compared between the pre-COVID-19 period (July 2018 to March 2020) and the COVID-19 period (April 2020 to December 2021) for both community and hospital settings. The chi-square test was used to determine if the differences in proportions were significantly different.

## Results

In the 21 months before the COVID-19 restrictions were put in place in Ontario, overall blood culture positivity rates in the community and in hospitals were 2.8% and 8.06%, respectively. During the 21 months of COVID-19 restrictions, overall blood culture positivity rates for the community remained the same but significantly increased (approximately three-fold; *p*=0.00E-00) for hospitals as compared to the preceding pre-pandemic period (**Table 1** and **Table 2**).

**Table 1:** Blood culture positivity rates in community settings by bacterial pathogens

Organisms	Pre-COVID-19 period^a^		COVID-19 period^a^		*p*-value^b^
	n	%	n	%	
Total blood cultures	32,411	100.00	25,860	100.00	-
All organisms	907	2.80	687	2.66	0.2971
CoNS	275	0.85	247	0.96	0.1746
*Escherichia coli*	118	0.36	69	0.27	0.0392
Viridans streptococci	97	0.30	97	0.38	0.1145
*Salmonella* spp.	89	0.27	13	0.05	0.0000
*Staphylococcus aureus*	57	0.18	32	0.12	0.1094
Enterococci	41	0.13	66	0.26	0.0003
*Klebsiella* spp.	38	0.12	37	0.14	0.3875
Other streptococci	15	0.05	15	0.06	0.5354
*Pseudomonas* spp.	12	0.04	4	0.02	0.1187
Yeast	10	0.03	13	0.05	0.2410
*Streptococcus pneumoniae*	5	0.02	5	0.02	0.7205
Other	41	0.13	52	0.20	0.0250
Anaerobe	56	0.17	26	0.10	0.0208
ESBL/AmpC	35	0.11	11	0.04	0.0052
SPICE	34	0.10	7	0.03	0.0004
MRSA	18	0.06	2	0.01	0.0020

Abbreviations: AmpC, AmpC beta-lactamases; CoNS, coagulase-negative staphylococci; ESBL, extended-spectrum beta-lactamase; MRSA, methicillin-resistant *Staphylococcus aureus*; SPICE, *Serratia*, *Pseudomonas*, indole-positive *Proteus*, *Citrobacter*, and *Enterobacter*

^a^ Pre-COVID-19 period: July 2018 to March 2020; COVID-19 period: April 2020 to December 2021

^b^ *p*-value obtained from chi-square tests

**Table 2:** Blood culture positivity rates in the hospitals by bacterial pathogens

Organisms	Pre-COVID-19 period^a^		COVID-19 period^a^		*p*-value^b^
	n	%	n	%	
Total blood cultures	88,170	100.00	42,665	100.00	-
All organisms	7,105	8.06	10,197	23.90	0.00E-00
*Escherichia coli*	1,410	1.60	2,026	4.75	1.6E-244
CoNS	1,045	1.19	1,698	3.98	6.6E-240
*Staphylococcus aureus*	860	0.98	1,200	2.81	3.3E-138
Other streptococci	461	0.52	593	1.39	8.88E-61
*Klebsiella* spp.	455	0.52	745	1.75	4E-106
Enterococci	424	0.48	648	1.52	6.98E-85
Viridans streptococci	245	0.28	376	0.88	4.02E-50
*Streptococcus pneumoniae*	221	0.25	176	0.41	6.03E-07
Yeast	182	0.21	255	0.60	1.34E-30
*Pseudomonas* spp.	170	0.19	286	0.67	5.89E-43
*Proteus mirabilis*	164	0.19	224	0.53	4.03E-26
*Salmonella* spp.	27	0.03	39	0.09	4.43E-06
Other	459	0.52	624	1.46	1.48E-69
Anaerobe	260	0.29	420	0.98	1.89E-59
ESBL/AmpC	182	0.21	171	0.40	2.1E-10
SPICE	215	0.24	348	0.82	1.22E-49
MRSA	507	0.58	539	1.26	3.06E-39

Abbreviations: AmpC, AmpC beta-lactamases; CoNS, coagulase-negative staphylococci; ESBL, extended-spectrum beta-lactamase; MRSA, methicillin-resistant *Staphylococcus aureus*; SPICE, *Serratia*, *Pseudomonas*, indole-positive *Proteus*, *Citrobacter*, and *Enterobacter*

^a^ Pre-COVID-19 period: July 2018 to March 2020; COVID-19 period: April 2020 to December 2021

^b^ *p*-value obtained from chi-square tests

**Figure 1:**
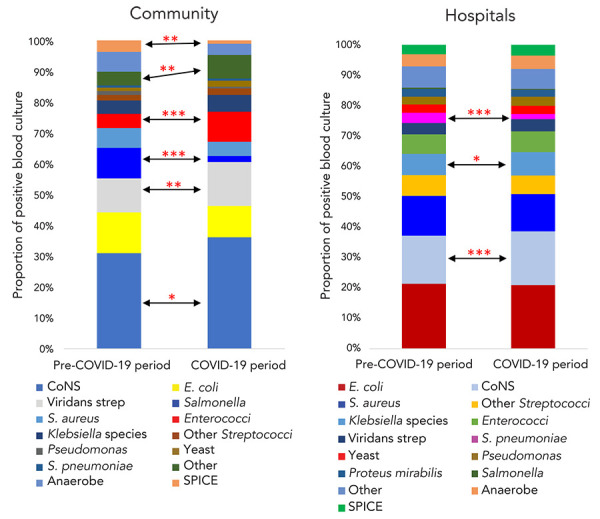
Relative proportion of pathogens recovered from positive blood cultures from the community or hospitals during the COVID-19 period compared to the pre-COVID-19 period^a^ Abbreviations: CoNS, coagulase-negative staphylococci; COVID-19, coronavirus disease 2019; *E. coli*, *Escherichia coli*; *S. aureus*, *Staphylococcus aureus*; *S. pneumoniae*, *Streptococcus pneumoniae*; SPICE, *Serratia*, *Pseudomonas*, indole-positive *Proteus*, *Citrobacter*, and *Enterobacter*; viridans strep, viridans streptococci ^a^ *p*-values calculated from two proportion Z-test; *p≤0.05; **p≤0.001; ***p≤0.0001

During the pre-pandemic period, the most frequently isolated bacterial species in blood cultures from the community were coagulase-negative staphylococci (CoNS), *E. coli*, viridans streptococci, *Salmonella* spp., *Staphylococcus aureus*, and *Enterococcus* spp. Both *S. pneumoniae* and *H. influenzae* were rarely isolated in BSIs from the community, perhaps reflecting widespread vaccination coverage for both species in Ontario. For the community, isolation rates of most bacterial species remained the same or changed very little during the COVID-19 pandemic, except in the cases of *Enterococcus* spp. and *Salmonella* spp. The rates of *Enteococcus* spp. increased about two-fold (*p*=0.0003) during the COVID-19 pandemic. The reason for this is not clearly understood, but may be attributed to changes in gut microbiome favouring *Enterococcus* spp. and increased intestinal permeability in COVID-19 patients, which have been recently described ((4)). On the other hand, the rates of *Salmonella* spp. in BSIs declined drastically (*p*=0.0000) in the community, which is likely associated with travel restrictions and physical distancing during the COVID-19 pandemic. Perhaps for the same reasons, the rates of antibiotic-resistant organisms (AROs) such as *Serratia*, *Pseudomonas*, indole-positive *Proteus*, *Citrobacter*, and *Enterobacter* (SPICE) organisms; ESBL/AmpC-producing Enterobacterales; and MRSAs also significantly (*p*<0.05) decreased in the community ((5)). Among the positive blood cultures from the community, the relative proportions of several bacterial species changed significantly during the COVID-19 pandemic. The proportions of CoNS, viridans streptococci, and *Enterococcus* spp. increased significantly (*p*≤0.05), while the proportions of *Salmonella* spp. and SPICE organisms decreased significantly (*p*≤0.001) (**Figure 1**).

For hospitals, the most frequently isolated bacterial species during the pre-COVID-19 period were *E. coli*, CoNS, *S. aureus*, other streptococci, *Klebsiella* spp., and *Enterococcus* spp. The isolation rates for all organism groups, including AROs, increased significantly (two to three-fold) during the COVID-19 pandemic, even though the total number of blood cultures was less than half than that reported during the pre-pandemic period. These results are consistent with higher incidence rates of hospital onset BSIs in other populations as well ((6–8)) and may be related to a higher rate of admission of COVID-19 patients to intensive care units. In the hospitals, the relative proportions of pathogens recovered from positive blood cultures were not significantly different for most pathogens, except for a significant increase in the proportion of positive blood cultures with CoNS (*p*≤0.0001) and a significant decrease in the proportion of positive blood cultures with *S. pneumoniae* (*p*≤0.0001) (Figure 1). A small but significant (*p*≤0.05) increase in the proportion of positive blood cultures with *Klebsiella* spp. was also noted during the COVID-19 period.

## Discussion

### Limitations

This study has several limitations. Although the study shows blood culture positivity rates for a representative Ontario population, it does not represent the accurate incidence of BSIs in Ontario because data were analyzed based on unique specimen accession numbers instead of patient identifiers. Also, because records of hospital admission dates were not available, the count of blood cultures received from hospitals may include a fraction that was community-acquired. It is likely that a small proportion of positive blood cultures, most commonly with CoNS and viridans streptococci, were reported as potential contaminants. However, this data could not be retrieved from the LifeLabs blood culture database.

## Conclusion

The blood culture data on overall and species-wise positivity rates for a large representative population suggest that there were shifts in BSI epidemiology in Ontario during the COVID-19 pandemic, both in hospitals and in the community.
